# Properties of SS304 Modified by Nickel–Cobalt Alloy Coating with Cauliflower-Shaped Micro/Nano Structures in Simulated PEMFC Cathode Environment

**DOI:** 10.3390/nano12121976

**Published:** 2022-06-09

**Authors:** Junji Xuan, Yueren Liu, Likun Xu, Yonglei Xin, Lili Xue, Li Li

**Affiliations:** 1 College of Materials Science and Chemical Engineering, Harbin Engineering University, Harbin 150001, China; xuanjunji@gmail.com (J.X.); xuelili@hrbeu.edu.cn (L.X.); lili_heu@hrbeu.edu.cn (L.L.); 2 State Key Laboratory for Marine Corrosion and Protection, Luoyang Ship Material Research Institute, Qingdao 266237, China; liuyueren@mail.nwpu.edu.cn (Y.L.); xinyl@sunrui.net (Y.X.)

**Keywords:** bipolar plates, stainless steel, PEMFC, Ni–Co, hydrophobicity, electrodeposition, corrosion resistance, conductivity, wettability

## Abstract

This study presents the corrosion behavior and surface properties of SS304 modified by electrodeposited nickel–cobalt (Ni–Co) alloy coating with cauliflower-shaped micro/nano structures (Ni–Co/SS304) in the simulated PEMFC cathodic environment. The hydrophobicity of the as-prepared Ni–Co alloy coating can be improved simply by low-temperature annealing. The morphology and composition of the Ni–Co/SS304 were analyzed and characterized by SEM, EDS, XRD, and XPS. The polarization, wettability, and ICR tests were respectively conducted to systemically evaluate the performance of Ni–Co/SS304 in the simulated PEMFC cathode environment. As revealed by the results, the Ni–Co/SS304 can maintain its hydrophobicity under hot-water droplets as high as 80 °C and demonstrates higher conductivity than the bare SS304 substrate before and after polarization (0.6 V vs. SCE, 5 h), which is of great significance to improve the surface hydrophobicity and conductivity of bipolar plates.

## 1. Introduction

Facing the energy crisis, the development of new energy, especially hydrogen energy, is becoming more and more urgent [1,2]. Proton exchange membrane fuel cell (PEMFC) is a promising hydrogen energy utilization device, in which bipolar plates are the crucial component for electron transfer and should possess high conductivity [3]. Meanwhile, PEMFC operates in a special environment (weak acidity, 60~80 °C, and the solution contains F^−^ and SO_4_^2−^), which requires the bipolar plates to have good corrosion resistance and low wettability [4,5,6,7,8]. The performance of bipolar plates influences the efficient, safe, and stable operation of PEMFC [9,10,11,12]. For example, the conductivity of bipolar plates affects the working efficiency of PEMFC, while the corrosion may initiate pitting (mainly for metal bipolar plates). High surface hydrophilicity of bipolar plates will retain water, block the flow field and aggravate local corrosion. However, the existing bipolar plate materials have many disadvantages. For example, graphite is fragile and expensive to process, aluminum demonstrates weak corrosion resistance, nickel and titanium show high material costs, and composite materials often exhibit unsatisfactory conductivity and mechanical properties [13,14,15]. In contrast, 304 stainless steel (SS304) has been widely studied as an excellent candidate material for bipolar plates because of its good and balanced material cost, conductivity, processability, and corrosion resistance [14,16,17,18]. Nevertheless, the passivation of stainless steel will cause its surface conductivity and wettability to decrease significantly, which cannot meet the application requirements. Therefore, surface modification is necessary before stainless steel is used as bipolar plates [19,20,21,22,23,24,25].

There are many studies on surface modification of stainless steel, including SS304 [19,26,27,28,29]. However, most modification methods need special devices, and the processing cost is very high. Nickel has strong corrosion resistance, and one of the main reasons for the excellent corrosion resistance of stainless steel is the addition of nickel components. In fact, nickel-based coatings prepared on bipolar plates show good properties. Feng et al. [30] prepared a nickel-rich layer on the SS316L surface by ion implantation technology, which showed that after different doses of nickel ion implantation, the passivation current density and the interfacial contact resistance (ICR) of SS316L clearly decreased. Through ion implantation technology, Feng et al. [31] also demonstrated that with the increase in nickel and chromium content (especially nickel) on the surface, the thickness of the passive film formed on SS316L decreased significantly. However, ion implantation technology still exhibits high requirements for the equipment and operating conditions. In contrast, due to the simple process and equipment, nickel plating on the metal surface not only has low processing costs, but can also effectively improve the corrosion resistance of the substrate [32]. However, the performance of nickel plating with single-component has its limitations, and it is an important way to improve the comprehensive properties of the plating by introducing other alloy elements into the plating and co-depositing. At present, Ni–P, Ni–Mo–P, Ni–Co–P, and Ni–Au–P have already been used to modify the surface of aluminum alloy bipolar plates, and the application of nickel-based alloy coatings can effectively improve the corrosion resistance and reduce the ICR of aluminum alloy bipolar plates [33,34,35,36,37,38].

Nickel–cobalt (Ni–Co) alloy coating is an excellent nickel-based alloy coating. Ni and Co can form the dense Ni–Co alloy solid solution, thus resulting in outstanding structural stability and corrosion resistance, and also usually demonstrating better mechanical properties than the other nickel-based alloy coatings [39,40]. For example, Bakhit et al. [41] had prepared and studied electrodeposited Ni–Co alloy coatings with cobalt content ranging from 0 to 45 wt.% on Cu plates, and the microhardness of the coating increased with the Co content in the alloy coatings. Meanwhile, the Ni–Co coatings with different Co contents showed good corrosion resistance in 3.5 wt.% NaCl solution. In particular, the manufactured metal bipolar plates at present are very thin to reduce the material cost, and the high mechanical strength of the coating can help the bipolar plates play a better role in structural support, while the high corrosion resistance can prevent pitting corrosion from causing perforation of the bipolar plates and potential safety hazards of the PEMFC. Meanwhile, the Ni–Co alloy coatings usually have high Ni contents (>50 wt.%), which is beneficial to reduce the ICR of stainless steel bipolar plates. Moreover, in previous studies, it has been found that the improvement of surface hydrophobicity by nickel-based alloy coatings is very limited, and the water contact angle (WCA) can only be increased to around 110° [42]. However, the Ni–Co coating prepared by Khorsand et al. [43] through electrodeposition had excellent hydrophobicity after being exposed to air for two weeks, showing WCA of about 158°, which is much higher than the nickel-based alloy coatings mentioned above, and it is also of great significance to improve the surface hydrophobicity of bipolar plates. In contrast, the traditional hydrophobicization strategy, i.e., reducing the surface wettability through the modification of low surface energy substances, usually requires the use of non-conductive organic substances, which will seriously reduce the surface conductivity of bipolar plates [44].

Although Ni–Co coatings show so many advantages, the performance of Ni–Co coated SS304 bipolar plates in PEMFC environments has not been systematically evaluated. Meanwhile, the wettability of bipolar plates coated with nickel-based coatings in hot-water environments or after service has not been studied, which is of great significance for the practical applications of bipolar plates. However, the surface structure of the Ni–Co coating prepared by Khorsand et al. [43] has many pores, which usually displays low mechanical strength, and the treatment time (exposure to air) is also too long, so the structure and process of the coating need to be further optimized when applied to bipolar plates. Liu et al. [45] found that the metallic surfaces prepared by electrodeposition can obtain high hydrophobicity by annealing at 65 °C for 12 h without using low surface energy substances, which can greatly reduce the time of surface treatment and inspire us to apply this process to the surface modification of bipolar plates. It is expected that the treatment strategy of low-temperature annealing may lead to the unique evolution of the properties (especially wettability) of the nickel-based alloy coated bipolar plates, which would have important guiding significance for subsequent modification research. Therefore, in this paper, the Ni–Co alloy coating was prepared on the SS304 surface by galvanostatic electrodeposition, and high hydrophobicity of the modified surface (Ni–Co/SS304) was obtained by annealing at 60 °C in an oven. The morphology and composition of Ni–Co/SS304 were characterized by SEM, EDS, and XRD. The effect of annealing time on the wettability of the modified surface was researched, and the origin of the decrease in the surface wettability of the Ni–Co alloy coating after annealing was investigated via XPS analysis. Then, the corrosion behavior and surface properties (e.g., wettability and conductivity) of Ni–Co/SS304 in the simulated PEMFC cathodic environment were systematically studied.

## 2. Materials and Methods

### 2.1. Materials

SS304 with chemical composition (wt.%) of 18.58 Cr, 8.65 Ni, 1.32 Mn, 0.39 Si, 0.024 C, 0.017 P, 0.004 S, and Fe (balance) was purchased commercially and used in this work. All SS304 samples needed sandpaper grinding. Then, the ultrasonic cleaning with pure water, ethanol, and acetone was performed sequentially. Meanwhile, the solvents and reagents, i.e., acetone (99%), ethanol (99.7%), H_2_SO_4_ (98%), HF (40%), H_3_BO_3_ (99%), CoSO_4_·7H_2_O (98.5%), NiCl_2_·6H_2_O (97%), NiSO_4_·6H_2_O (98%), were used and purchased from China Sinopharm Chemical Reagent Co, Ltd. (Shanghai, China). The used ultrapure water was made in the laboratory.

### 2.2. Preparation of Electrodeposited Hydrophobic Ni–Co/SS304

**Figure 1:**
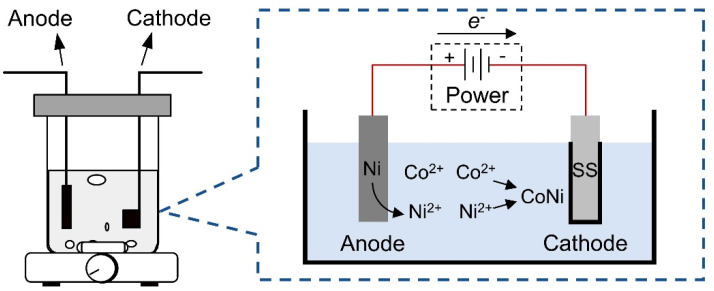
Schematic diagrams of the device and principle of electrodeposition of Ni–Co coating.

The Ni–Co/SS304 was prepared by galvanostatic deposition method (45 mA/cm^2^, 30 min) and referring to the literatures [41,46,47], and the Ni–Co coating prepared under this condition usually has excellent corrosion resistance and microhardness. The experimental setup and deposition principle are shown in Figure 1. A two-electrode system was used in the deposition process, which was as follows: the cathode (counter electrode) was SS304 (10 mm × 10 mm × 10 mm); the anode (working electrode) was a nickel plate (20 mm × 20 mm × 5 mm, ≥99.99 wt.% Ni). The composition of the plating solution (200 mL) was as follows: CoSO_4_ (30 g/L), NiCl_2_ (40 g/L), NiSO_4_ (220 g/L), and H_3_BO_3_ (30 g/L). The electrodeposition was conducted at 50 °C with magnetic stirring (800 rpm). After electrodeposition, the sample surface was cleaned with pure water, ethanol, and acetone in sequence, and then dried in vacuum. Afterwards, the Ni–Co/SS304 was annealed in an electric oven at 60 °C for 48 h to acquire high hydrophobicity.

### 2.3. Surface Analyses

The surface morphology of specimens was characterized by a scanning electron microscope (SEM, ULTRA 55, ZEISS, Oberkochen, Germany). The surface elemental composition was analyzed by an energy dispersive spectrometer (EDS, X-Max, Oxford Instruments, Abingdon, UK). The thickness of the electrodeposited coating was measured by a 3D measuring laser microscope (LEXT OLS4000, Olympus, Tokyo, Japan), after the coating is separated from the substrate by mechanical treatment. For characterizing the crystalline phase of the samples, an X-ray diffractometer (XRD, D8 ADVANCE, Bruker, Karlsruhe, Germany) with Cu Kα radiation as the beam was used. An X-ray photoelectron spectrometer (XPS, 250Xi, Thermo Fisher Scientific, Waltham, MA, USA) was used to reveal the changes in chemical element states before and after annealing. The sliding angle (SA) and WCA of the surfaces were recorded by employing a tensiometer (Attention Theta, KSV Instruments, Helsinki, Finland). In addition, WCA and SA were the averages of values of the three results obtained from different samples. The surface conductivity was evaluated by the ICR tests, which were conducted with reference to the literature [4,48,49], using the same experimental setup and test procedure as that in previous research [50]. The ICR tests were repeated 3 times and the average value was taken.

### 2.4. PEMFC Simulated Solutions

A solution close to the real operating condition of PEMFC was determined as the main simulated PEMFC cathode environment, namely a weakly acidic solution containing 1 × 10^−5^ M H_2_SO_4_ and 2 ppm F^−^, the F^−^ source was HF, and the solution temperature was 80 °C [51,52,53]. It should be noted that the simulated solution needed to be treated with air bubbling throughout the electrochemical experiments. Meanwhile, with the technical progress of PEM and gas diffusion layer and the continuous improvement of battery design in recent years, the PEMFC anode environment has great uncertainty and complexity, so it will not be discussed in this paper.

### 2.5. Electrochemical Tests

Electrochemical measurements were performed using an electrochemical workstation (PARSTAT 2273, Princeton Applied Research, PA, USA). The following three-electrode system was used: the tested sample was the working electrode, the counter electrode was a platinum-coated niobium wire, and the reference electrode was a saturated calomel electrode (SCE). Before the electrochemical tests, open circuit tests were carried out (2 h) for obtaining a stable open circuit potential (OCP). The potentiodynamic polarization experiments were scanned from −0.6 V (vs. OCP) to 1.2 V (vs. SCE) at 2 mV/s. As for the potentiostatic polarization tests, the specimens were polarized at 0.6 V (vs. SCE) for 5 h in a simulated PEMFC cathode environment.

## 3. Results and Discussion

### 3.1. Morphology and Composition

**Figure 2:**
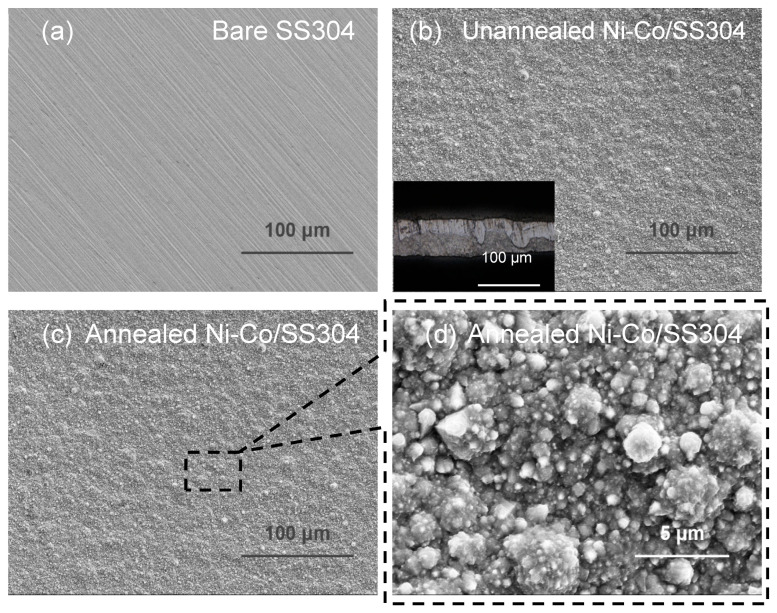
SEM surface morphology of different surfaces: (**a**) bare SS304; (**b**) unannealed Ni–Co/SS304, and the illustration in (**b**) is the cross-section of Ni–Co coating photographed by a 3D measuring laser microscope; (**c**,**d**) annealed Ni–Co/SS304.

The SEM surface morphology of SS304 samples before and after treatment is displayed in Figure 2. As shown in Figure 2a, the bare SS304 surface is flat and clean, and the textures of scratches observed on it are mainly caused by sandpaper grinding. After electrodeposition, as demonstrated in Figure 2b, the scratches on the SS304 surface disappear and are completely covered by the coating, which indicates that the coating exhibits high compactness and thickness. Meanwhile, there are many micro/nano protrusions on the coating surface. The thickness of the coating was measured to be 55.9 ± 3.1 μm, as shown in the illustration in Figure 2b. In addition, the coating grows closely with the SS304 substrate, demonstrating strong adhesion and high mechanical strength. According to the determination method of metal layer adhesion in GB/T 40262-2021, the adhesion of the prepared coating is grade 5. It is known that Liu et al. [45] prepared a series of metal surfaces with high hydrophobicity by electrodeposition, without using low surface energy substance. It had been proved that the main reason for the high hydrophobicity was the adsorption of airborne hydrocarbons, which could reduce the surface energy of metal surfaces. Meanwhile, their study revealed that the adsorption of airborne hydrocarbons on metal surfaces was almost inevitable, and the increase in ambient temperature could accelerate this process. Then, the electrodeposited SS304 surface was annealed in the oven at 60 °C to rapidly improve the surface hydrophobicity. However, the surface morphology of the sample (Figure 2c) after annealing had no obvious differences from that before annealing. High-magnification observation (Figure 2d) shows that the micro/nano convex structures grown on the coating are mostly cone-shaped or cauliflower-shaped, with different sizes, mainly in the range of 0.3~5 μm.

The EDS composition information of SS304 modified by electrodeposition before and after annealing is listed in Table 1. The surface elements of the electrodeposited sample are Ni, Co, C, and O, while the main elements are Ni and Co, and their atomic content ratio is close to 1:1, indicating that the coating prepared on the SS304 surface is a Ni–Co alloy. After annealing, the elements of the Ni–Co alloy coating are still the same, but the relative contents of C and O increase, which indicates that the oxidation degree of the Ni–Co alloy coated surface increases after treatment, and many organic substances may be adsorbed on the coating surface [43,45,54]. EDS mapping (Figure S1) also shows that the distribution of Ni and Co elements is very uniform, indicating that the Ni–Co coating is likely to be composed of a single phase. However, the surface morphology of the annealed sample does not change.

**Figure 3:**
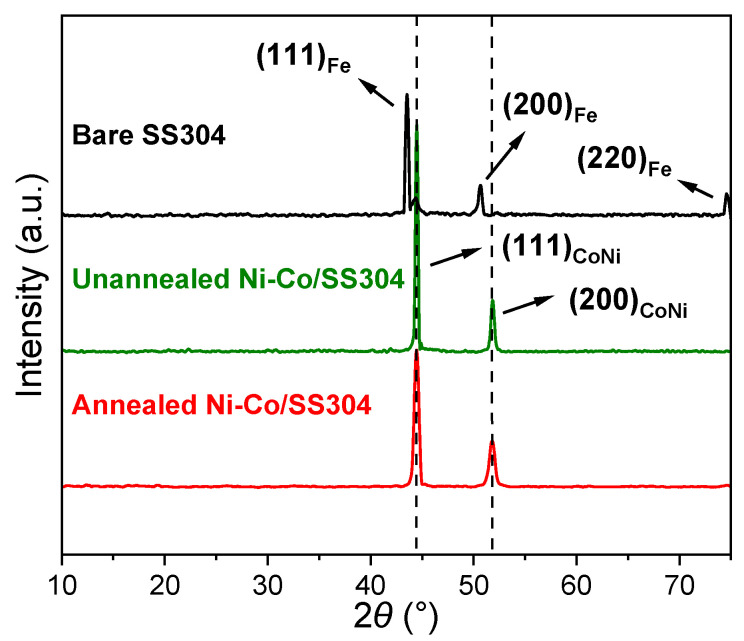
XRD patterns of bare SS304, unannealed Ni–Co/SS304, and annealed Ni–Co/SS304.

Figure 3 shows the XRD patterns of bare SS304 and Ni–Co/SS304 before and after annealing. The diffraction peaks of bare SS304 appear at 43.6, 50.8 and 74.7°, corresponding to the (111), (200) and (220) crystal planes of its austenite structure (Fe) [55]. As for the XRD patterns of the Ni–Co/SS304 samples, the diffraction peaks of the SS304 substrate cannot be observed, but new diffraction peaks appear at 44.4 and 51.9°, which are consistent with the peaks that come from the (111) and (200) crystal planes of CoNi (1/1) solid solution (ICDD-PDF 97-010-8308) with a face-centered cubic structure. The diffraction peak positions of the XRD patterns of Ni–Co/SS304 before and after annealing do not demonstrate significant differences, which indicates that the low-temperature annealing treatment does not affect the phase structure of the Ni–Co alloy coating.

### 3.2. Surface Wettability of Electrodeposited Ni–Co Alloy Coatings

**Figure 4:**
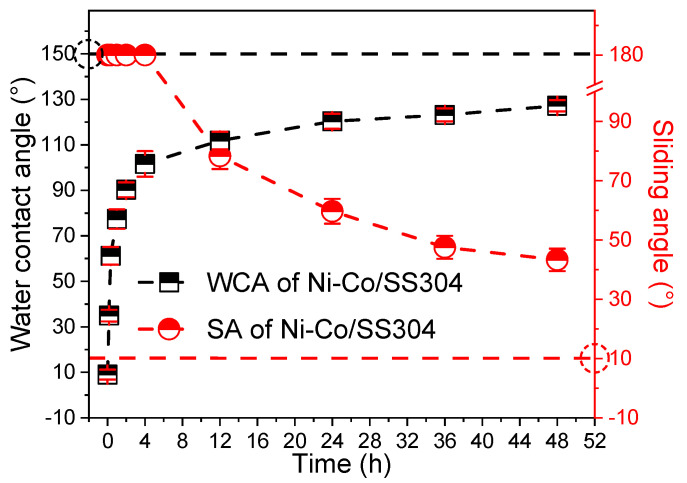
WCA and SA changes in Ni–Co/SS304 surface with annealing time.

The surface wettability change in Ni–Co/SS304 with annealing time is shown in Figure 4. The unannealed Ni–Co/SS304 surface exhibits superhydrophilicity (WCA < 10°). With the extension of annealing time, the WCA of the Ni–Co/SS304 surface gradually increases, while the SA gradually decreases. When the annealing time is longer than 2 h, the surface of Ni–Co/SS304 becomes hydrophobic. The Ni–Co/SS304 surface needs to be annealed for more than 48 h to obtain stable WCA and SA, and the final stable WCA and SA values are about 127.1° and 43.3°, respectively. If the annealing time is extended to two weeks, the WCA and SA will still increase and decrease, respectively, but the changes are very small. After two weeks, the WCA would not exceed 135°. Considering the time cost and benefit, the hydrophobicity of the Ni–Co coating generated within two days is acceptable. In brief, the above results reveal that low-temperature annealing treatment can significantly improve the hydrophobicity of the Ni–Co/SS304 surface.

**Figure 5:**
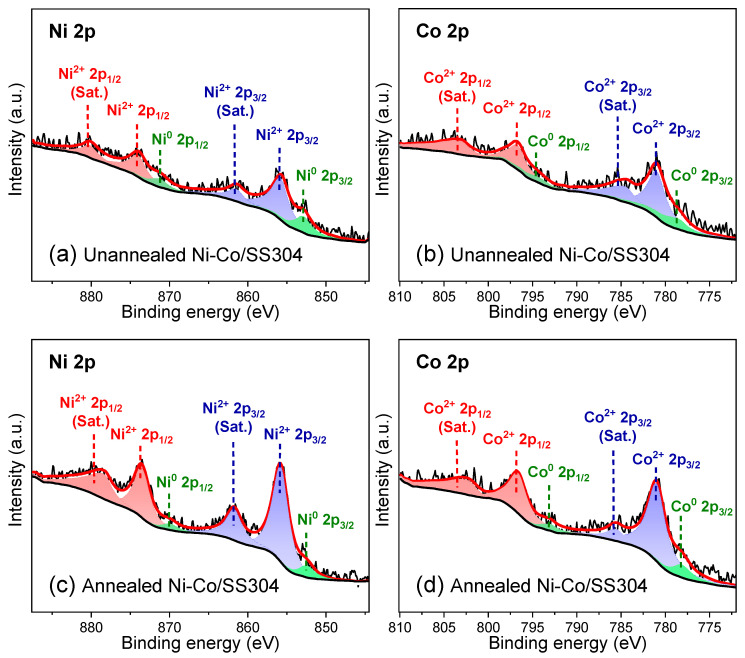
XPS spectra of Ni 2p and Co 2p of Ni–Co/SS304: (**a**,**b**) unannealed; (**c**,**d**) annealed.

**Figure 6:**
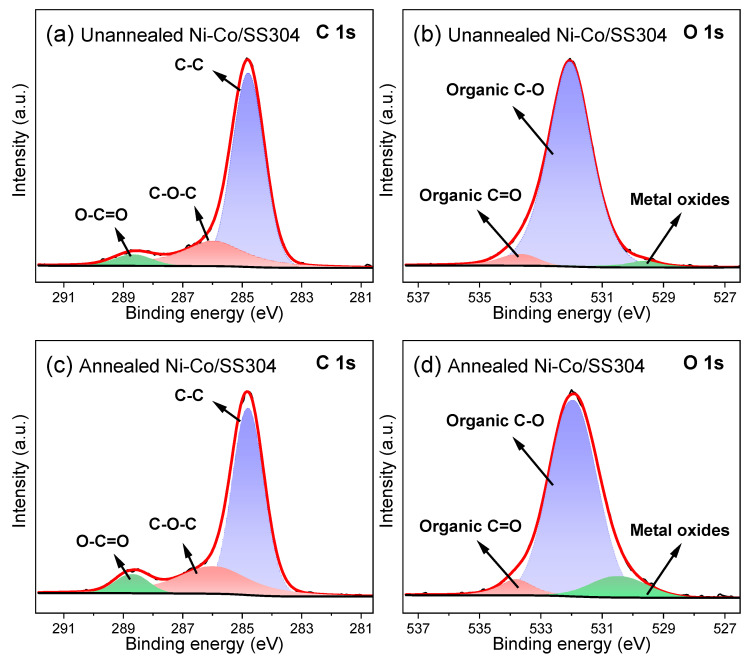
XPS spectra of C 1s and O 1s of Ni–Co/SS304: (**a**,**b**) unannealed; (**c**,**d**) annealed.

To find out the essential reason for the increase in hydrophobicity of the Ni–Co/SS304 surface caused by annealing treatment, XPS analysis was conducted (Figure 5 and Figure 6). Figure 5a,b display the XPS spectra of Ni 2p and Co 2p orbitals of the unannealed Ni–Co/SS304 surface, which can be divided into six characteristic peaks, belonging to Ni^2+^, Ni^0^, Co^2+^, and Co^0^, respectively. These peaks are related to the existence of Ni and Co elements with different valence states in NiO, CoO, and CoNi solid solutions [43]. Figure 5c,d show that after annealing treatment, the same characteristic peaks are also found in the XPS spectra of Ni 2p and Co 2p orbitals of Ni–Co/SS304, which should be derived from the same elements and valence states. However, after annealing, the relative atomic content of the surface metal state of the Ni–Co alloy coating in the total content of Ni and Co decreased from about 30.49% and 28.77% to about 11.36% and 14.20%, respectively. The above results show that annealing increases the degree of oxidation of the Ni–Co alloy coating surface. This is consistent with the result of EDS. However, due to the high degree of surface oxidation before annealing, the oxidation caused by annealing is quite limited. Therefore, it is unlikely that the significant decrease in surface wettability of the Ni–Co alloy coating is simply caused by the increase in surface oxidation degree.

According to a common theory, as also mentioned above, it is believed that the adsorption of airborne hydrocarbons (usually with low surface energy, mostly volatile organic compounds) will lead to an increase in surface hydrophobicity [43,45,56]. Therefore, as shown in Figure 6, the XPS spectra of the C 1s and O 1s orbitals of the Ni–Co alloy coating were further analyzed. According to the XPS spectra of the C 1s orbital in Figure 6a,c, the characteristic peaks of unannealed Ni–Co/SS304 at 286 eV (C-O-C) and 288.7 eV (O-C=O) are relatively low, while these characteristic peaks are significantly enhanced after the Ni–Co alloy coating is annealed, indicating that only a small amount of organic matter may be attached to the Ni–Co alloy coating surface before annealing, and more organics are adsorbed on the surface after the annealing treatment [43]. As shown in Figure 6b,d, the characteristic peak of C=O in the O 1s orbital of the Ni–Co alloy coating after annealing is also enhanced compared with that before annealing, which also confirms the increase in organic adsorption on the surface. In addition, after annealing, the characteristic peaks of metal oxides at 529~530 eV in Figure 6d rise notably, which is also strong evidence for the increase in oxidation degree on the Ni–Co alloy coating surface [57]. The above results reveal that the transition of the Ni–Co alloy coating from hydrophilicity to hydrophobicity is caused by the adsorption of airborne hydrocarbons. This is of great guiding significance to the improvement of the hydrophobicity of SS304 bipolar plates by surface modification.

### 3.3. Corrosion Behavior of Ni–Co/SS304 in the Simulated PEMFC Cathodic Environment

**Figure 7:**
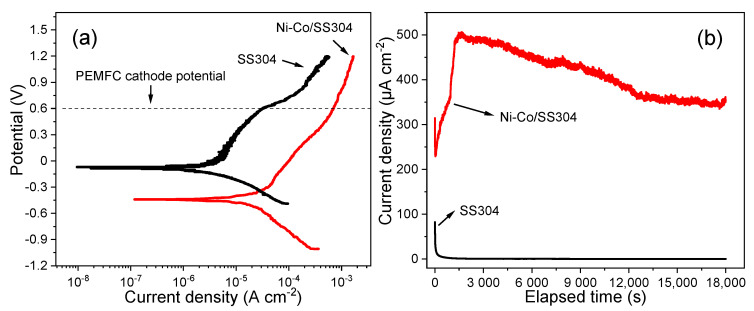
The polarization curves of SS304 and Ni–Co/SS304 in the simulated PEMFC cathodic environment: (**a**) potentiodynamic polarization curves, (**b**) potentiostatic polarization curves (0.6 V, 5 h).

The corrosion behavior of Ni–Co/SS304 (which hereafter refers to the annealed sample) in the simulated PEMFC cathodic environment was investigated by potentiodynamic and potentiostatic polarizations. As shown in Figure 7a, the cathodic polarization curves of bare SS304 and Ni–Co/SS304 exhibit similar behaviors. However, at the same potential, the current density of Ni–Co/SS304 is lower than that of bare SS304, indicating that the cathodic reaction of SS304 is inhibited after being modified by the Ni–Co alloy coating. Meanwhile, the anodic polarization behaviors of bare SS304 and Ni–Co/SS304 are also similar with a few differences. The active-passive transition process is not observed in the two anodic polarization curves, which is related to their spontaneous passivation behavior in the simulated PEMFC solution. With the polarization potential rising, the anodic current densities of the two samples increase slowly and proportionally, and the polarizability gradually increases, which may be caused by the dynamic dissolution and growth process of the surface passive film. When it reaches a certain potential, the current density of bare SS304 increases rapidly, which indicates that the passive film begins to breakdown, and the corresponding potential is breakdown potential (*E*_b_). However, the current density of Ni–Co/SS304 still increases approximately linearly, and there is no obvious passive-transpassive transition process. Above −0.3 V, although the anode current density of Ni–Co/SS304 increases slowly, it is much higher than that of bare SS304. The above anodic polarization behavior of Ni–Co/SS304 is usually called pseudo-passivation [58].

Table 2 lists the polarization parameters of the potentiodynamic polarization curves of bare SS304 and Ni–Co/SS304. Ni–Co/SS304 demonstrates lower corrosion potential and higher corrosion current density compared with bare SS304, which indicates that the corrosion resistance of Ni–Co/SS304 is lower than that of the bare substrate, showing higher corrosion tendency. Meanwhile, at the typical cathode working potential of PEMFC (0.6 V vs. SCE), both bare SS304 and Ni–Co/SS304 are in corrosion state, but the current density of Ni–Co/SS304 is much higher than that of bare SS304.

Then, 5 h potentiostatic polarization (0.6 V vs. SCE) was conducted to simulate the long-term PEMFC working condition. As shown in Figure 7b, the current density of both bare SS304 and Ni–Co/SS304 decreases rapidly with time in the initial stage, which may be related to the rapid passivation of the surface of SS304, and the oxide film formed on the surface of the Ni–Co alloy coating before polarization. As the polarization continues, the current density of bare SS304 is still decreasing gradually and slowly, while the current density of Ni–Co/SS304 begins to increase rapidly, and it reaches a maximum value of about 500 μA/cm^2^ after 1700 s. Then, as the polarization time increases, the current density of Ni–Co/SS304 begins to decrease steadily and finally stabilizes at about 350 μA/cm^2^, which is larger than the final stable current density of bare SS304 (0.291 μA/cm^2^). The increase in the current density of Ni–Co/SS304 is related to the damage of the protective film formed on the surface before polarization, while the decrease in the current density is associated with the formation of the new oxide film. The above results show that in the simulated PEMFC cathodic environment, the oxide film formed on the Ni–Co/SS304 surface can inhibit further corrosion, but the corrosion resistance is lower than that of the SS304 substrate.

### 3.4. Surface Properties of Ni–Co/SS304 in the Simulated PEMFC Cathodic Environment

**Figure 8:**
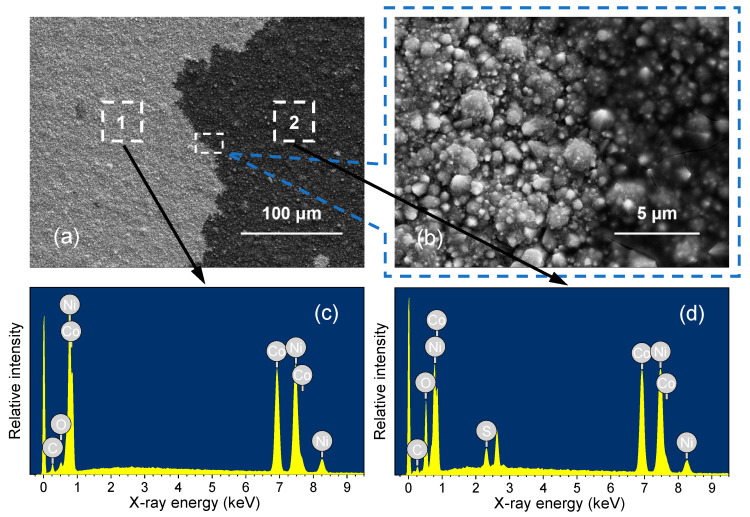
The SEM surface morphology at low (**a**) and high (**b**) magnification and EDS spectra of area 1 (**c**) and area 2 (**d**) with different morphologies of Ni–Co/SS304 after polarization (0.6 V, 5 h) in the simulated PEMFC cathodic environment.

The surface morphology, composition, wettability, and conductivity were characterized to further explore the surface property changes in Ni–Co/SS304 in the simulated PEMFC cathodic environment. The SEM images of the Ni–Co/SS304 surface after potentiostatic polarization (0.6 V, 5 h) are shown in Figure 8, and the sample surface shows obvious local corrosion. A large area of corrosion product (dark part) appears on the surface of polarized Ni–Co/SS304, while the cauliflower-shaped micro/nano structures still exist. At high magnification (Figure 8b), the observed surface morphology of the polarized sample has no obvious change compared with that before polarization in the area, where there is no corrosion product accumulation. However, in the dark area, the micro/nano structures of the polarized Ni–Co/SS304 surface are almost completely covered by corrosion products, showing masked morphology. In addition, many cracks also appeared in these areas, which may be related to the composition characteristics of the corrosion products.

Figure 8c,d show the EDS spectra of the two different regions on polarized Ni–Co/SS304, and the detailed compositions are listed in Table 3. The area without corrosion product accumulation (area 1) mainly contains Ni, Co, C, and O. Compared with the polarized Ni–Co/SS304 surface, in area 1, the relative content of Ni increases slightly, while the content of Co and C decreases. The content of C has been reduced to close to that before annealing treatment, but the content of O changes little. As shown in Figure 8d and Table 3, the area with corrosion product accumulation (area 2) contains Ni, Co, C, O, and S. In area 2, the relative content ratio of Ni/Co is similar to that of area 1, and the content of C is much lower, while the content of O is extremely high, and a small amount of S appears. The composition difference reveals that the corrosion rate of alloying elements in the Ni–Co alloy coating in these two areas is similar, and the Co dissolves preferentially. The hydrocarbons adsorbed on the Ni–Co/SS304 surface have been separated from the surface after polarization, which may lead to the increase in surface wettability.

**Figure 9:**
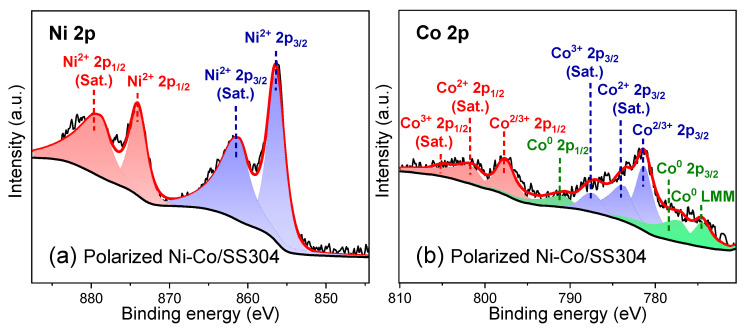

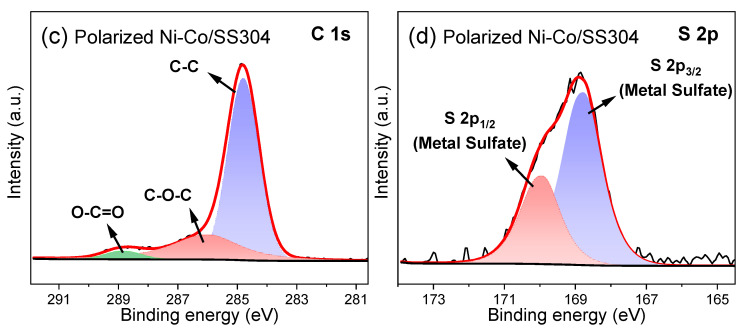
XPS spectra of Ni 2p, Co 2p, C 1s, and O 1s of Ni–Co/SS304 after polarization (0.6 V, 5 h) in the simulated PEMFC cathodic environment.

The XPS analysis was carried out to further understand the chemical composition of the oxide film formed on the surface after polarization and the types of possible corrosion products. As displayed in Figure 9, the XPS spectrum of the Ni 2p orbit of polarized Ni–Co/SS304 mainly shows the characteristic peaks of Ni^2+^ in NiO, while the component of metallic Ni disappears compared with that before polarization. In contrast, the component of the Co 2p spectrum of the polarized sample is very complicated, among which the split spin-orbit components of Co^0^, Co^2+^, and Co^3+^ can be found. The Co LMM Auger peak and the corresponding Co 2p split spin-orbit components represent the presence of metal Co on the surface, while the satellite features owing to 2+ and 3+ states of Co can sufficiently prove the existence of CoO and Co_3_O_4_. The characteristic peaks of C–O–C and O–C=O in the C 1s spectrum are reduced, which is equivalent to that of the sample before annealing. As for the S 2p spectrum, the closely spaced spin-orbit components at 168.77 and 169.93 eV belong to metal sulfates. The results reveal that the oxides and corrosion products formed on the surface are mainly NiO, CoO, Co_3_O_4_, and sulfates of Ni or Co. The corrosion resistance of the Ni–Co alloy coating is closely related to the formation of oxides of NiO, CoO, and Co_3_O_4_, which will further react with sulfuric acid in the simulated PEMFC solution at 0.6 V to form the corresponding sulfates. However, the appearance of metal Co indicates that the corrosion resistance of Co oxides is not as good as that of Ni oxides, and they are more likely to react with sulfuric acid or dissolve in the solution, which will lead to the exposure of metal components. Besides, the weakening of the peaks of C 1s components confirms the reduction in hydrocarbons adsorbed on the surface. Typically, the above chemical composition changes could lead to a significant increase in surface wettability, which will be verified by the wettability characterization.

**Figure 10:**
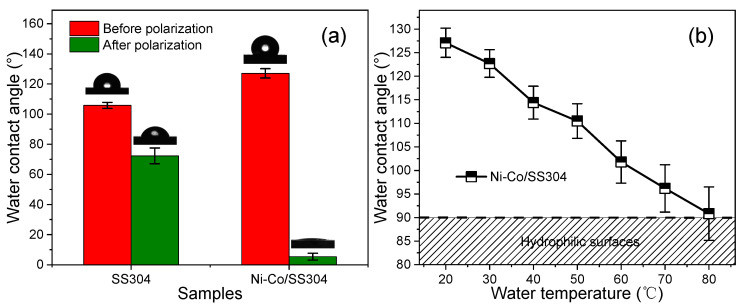
(**a**) WCAs of bare SS304 and Ni–Co/SS304 surfaces before and after polarization (0.6 V, 5 h); (**b**) WCAs of Ni–Co/SS304 surface under water droplets at different temperatures.

Figure 10a shows the WCA of bare SS304 and Ni–Co/SS304 surfaces before and after potentiostatic polarization (0.6 V, 5 h). The bare SS304 surface is hydrophobic (WCA = 106.2 ± 1.8°) and becomes hydrophilic (WCA = 71.8 ± 5.6°) after polarization. After electrodeposition of the Ni–Co alloy coating, the WCA of the Ni–Co/SS304 surface can be increased to 127.1 ± 3.1°. However, as predicted by the EDS and XPS results, its surface wettability also increases sharply and becomes superhydrophilic (WCA = 5.4 ± 2.3°) with polarization (0.6 V, 5 h), which is most likely caused by the desorption of surface hydrocarbons and the generation of hydrophilic corrosion products. As discussed, the increase in hydrophobicity of the Ni–Co/SS304 surface is mainly caused by the attachment of hydrocarbons. In addition, the working environment temperature of PEMFC is usually around 80 °C, so the hot water repellency of the bipolar plates is also very important. Figure 10b demonstrates the WCA of the Ni–Co/SS304 surface under water droplets at different temperatures. With the increase in water temperature, the WCA of the Ni–Co/SS304 surface decreases gradually, but when the water droplet temperature reaches 80 °C, its WCA is still greater than 90°. The above changes in WCA reveal that the electrodeposited Ni–Co alloy coating can improve the hydrophobicity of the bare SS304 surface, and even in the high-temperature PEMFC environment, the surface of Ni–Co/SS304 is still hydrophobic. Nevertheless, its hydrophobicity is still unstable and will be reduced by polarization, in a similar way to the SS304 substrate.

**Figure 11:**
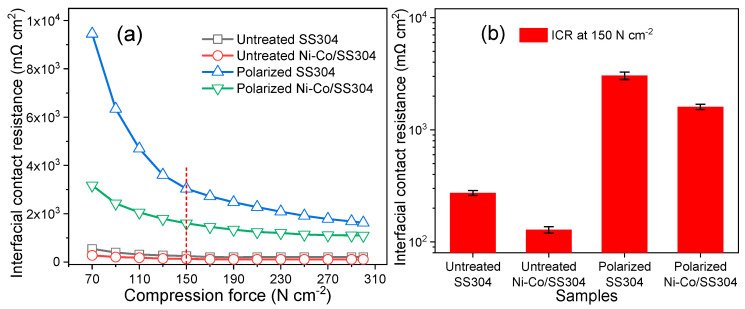
The interfacial contact resistance of SS304 and Ni–Co/SS304 before (untreated) and after polarization (0.6 V, 5 h) in the simulated PEMFC cathode environment: (**a**) ICR at various compression forces; (**b**) ICR at 150 N/cm^2^.

Figure 11 shows the ICR of bare SS304 and Ni–Co/SS304 surfaces before and after potentiostatic polarization (0.6 V, 5 h). The ICR values of all samples gradually decrease with the increase in the applied compression pressure. At the typical stacking compression pressure of 150 N/cm^2^ for bipolar plates, the ICR of polarized samples is much larger than that before polarization, which may be due to the formation of oxide films with poor conductivity on the surface after polarization. Meanwhile, the ICR values of SS304 before and after polarization are larger than that of the corresponding Ni–Co/SS304, which manifests that the surface conductivity of the electrodeposited Ni–Co alloy coating is better than that of the SS304 substrate. This may be attributed to the formation of more nickel oxides on the surface of Ni–Co alloy coating, and the ICR of nickel oxides is much lower than that of iron and chromium oxides formed on the SS304 substrate, as proved by the literature [30,31].

## 4. Conclusions

In summary, a Ni–Co alloy coating with cauliflower-shaped micro/nano structures was prepared on the SS304 surface by galvanostatic deposition to improve the performance of SS304 bipolar plates in the PEMFC environment. The main composition of the electrodeposited Ni–Co alloy coating was a CoNi (1/1) solid solution with a face-centered cubic structure, and the Ni–Co/SS304 exhibited high hydrophobicity after annealing, of which the origin of the increased hydrophobicity was related to the surface adsorption of airborne hydrocarbons. Meanwhile, the Ni–Co/SS304 could also maintain the hydrophobic state under the hot-water droplet up to 80 °C. In the simulated PEMFC cathodic environment, although the corrosion resistance of Ni–Co/SS304 was not as good as that of the SS304 substrate, the formation of a surface oxide film could still inhibit corrosion. The surface wettability of Ni–Co/SS304 increases in a similar way to the bare SS304 after potentiostatic polarization (0.6 V, 5 h), which was associated with the formation of hydrophilic oxides and needed special attention. In addition, the ICR values of Ni–Co/SS304 before and after potentiostatic polarization were lower than that of the bare SS304 substrate, showing better conductivity. Generally speaking, the electrodeposited Ni–Co alloy coating with cauliflower-shaped micro/nano structures can improve the hydrophobicity and surface conductivity of the SS304 substrate as bipolar plates. This research provides a new idea for improving the surface hydrophobicity of bipolar plates and lays a good theoretical basis for preparing high performance stainless-steel bipolar plates by electrodeposition modification.

## Figures and Tables

**Table 1:** Elements and contents (at.%) of Ni–Co/SS304 surface before and after annealing obtained by EDS.

Samples	Ni	Co	C	O
Unannealed Ni–Co/SS304	50.32	43.87	2.96	2.85
Annealed Ni–Co/SS304	49.39	42.85	3.71	4.05

**Table 2:** Polarization parameters derived from potentiodynamic polarization curves of SS304 and Ni–Co/SS304 shown in Figure 7a.

Samples	*E*_corr_ (V vs. SCE)	*E*_b_ (V vs. SCE)	*i*_corr_ (μA/cm^2^)	*i*_0.6_ V vs. SCE (μA/cm^2^)
SS304	−0.0702	0.6008	4.395	32.27
Ni–Co/SS304	−0.4413	-	13.81	703.3

**Table 3:** Elements and contents (at.%) of area 1 and area 2 with different morphologies on polarized Ni–Co/SS304 in Figure 8.

Polarized Ni–Co/SS304	Ni	Co	C	O
Area 1	51.27	42.15	2.60	3.97
Area 2	34.12	28.25	1.38	33.64

## Data Availability

The data that support the results of this study are available from the corresponding author upon reasonable request.

## Supplementary Materials

The following supporting information can be downloaded at: https://www.mdpi.com/article/10.3390/nano12121976/s1, Figure S1: EDS mapping of the surface elements of the cauliflower-shaped structure on the electrodeposited Ni–Co coating.
