# Supplementary material for: Properties of SS304 Modified by Nickel–Cobalt Alloy Coating with Cauliflower-Shaped Micro/Nano Structures in Simulated PEMFC Cathode Environment
Source: Nanomaterials (Basel). 2022 Jun 9;12(12):1976. doi: 10.3390/nano12121976 (PMC9228718; doi:10.3390/nano12121976)
Supplement: Supplementary file 1 [file nanomaterials-12-01976-s001.zip › nanomaterials-1737253-supplementary.pdf]

Supplementary materials

Properties of SS304 modified by nickel-cobalt alloy coating with cauliflower-shaped micro/nano structures in simulated PEMFC cathode environment

Junji Xuan [1,2], Yueren Liu [2], Likun Xu [1,2,*], Yonglei Xin [2], Lili Xue [1], Li Li [1]

[1] College of Materials Science and Chemical Engineering, Harbin Engineering University, Harbin 150001, P. R. China

[2] State Key Laboratory for Marine Corrosion and Protection, Luoyang Ship Material Research Institute, Qingdao 266237, P. R. China

[*] Correspondence: xulk@sunrui.net (L.X.)

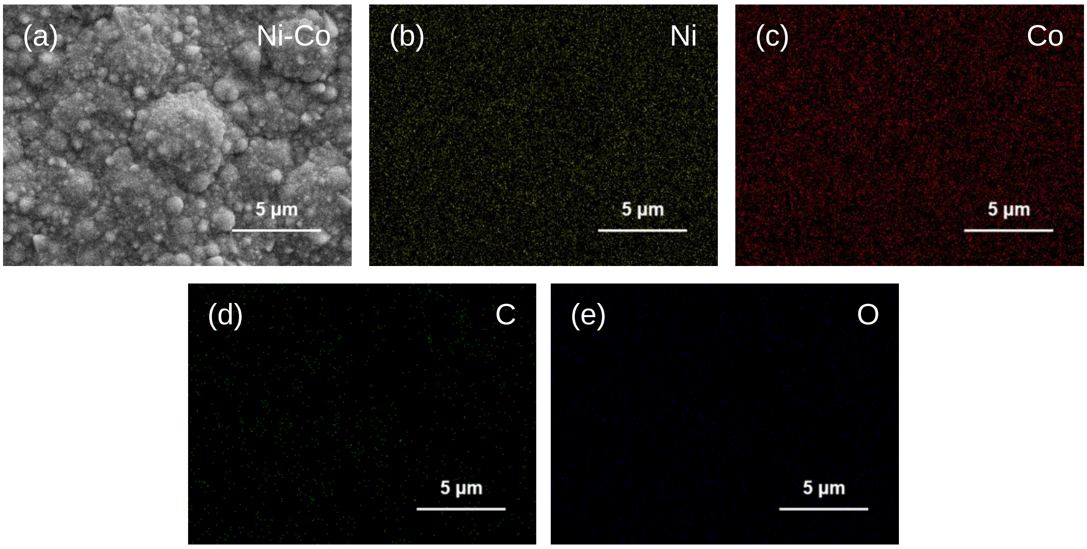

Figure S1. EDS mapping of the surface elements of the cauliflower-shaped structure on the electrodeposited Ni-Co coating. (a) Ni-Co; (b) Ni; (c) Co; (d) C; (e) O.
